# Is There an Association Between Chronic Obstructive Pulmonary Disease and Chronic Renal Failure?

**DOI:** 10.7759/cureus.26149

**Published:** 2022-06-21

**Authors:** Nikolaos Madouros, Sommer Jarvis, Amber Saleem, Evgenia Koumadoraki, Shayka Sharif, Safeera Khan

**Affiliations:** 1 Surgery, California Institute of Behavioral Neurosciences & Psychology, Fairfield, USA; 2 Anatomy/Cell Biology, California Institute of Behavioral Neurosciences & Psychology, Fairfield, USA; 3 Family Medicine, California Institute of Behavioral Neurosciences & Psychology, Fairfield, USA; 4 Pathology, California Institute of Behavioral Neurosciences & Psychology, fairfield, USA; 5 Internal Medicine, California Institute of Behavioral Neurosciences & Psychology, Fairfield, USA

**Keywords:** smoking, pulmonary disease, chronic kidney disease (ckd), chronic renal failure, copd: chronic obstructive pulmonary disease

## Abstract

Chronic obstructive pulmonary disease (COPD) is one of the most common diseases worldwide with its prevalence increasing with age. It is commonly comorbid with other diseases and managing patients could be difficult and expensive. Chronic kidney disease (CKD) is often present in COPD patients and may be underdiagnosed, especially if it is mild. This study intended to summarize recent findings showing the correlation between the two diseases. Studies were gathered that were published in the last 11 years, from 2010 to 2021. PubMed was used as the main source of data, but papers from the references of the included other sources were added for thoroughness. Observational studies on examining the prevalence and prognosis of comorbid COPD and CKD published in the English language were included. A higher prevalence of CKD in COPD patients was found in most studies; it was found that a higher risk of mortality is present if these diseases coexist. Further research is required and more extensive prospective studies are needed with matched control groups to support the correlation.

## Introduction and background

According to the Global Burden of Disease Study published in 2015, chronic obstructive pulmonary disorder (COPD) was responsible for the death of 3.2 million people with this number increasing from 1990 by 11.6%, mainly due to longer life expectancy and a larger population [1]. COPD is a preventable and treatable respiratory disease [2]. It is characterized by airflow obstruction that is not fully reversible. In the past, it was categorized as either chronic bronchitis or emphysema based on the predominant symptoms (dyspnea for emphysema and productive cough for chronic bronchitis) and was associated with inflammatory reaction against noxious particles or gases [2].

Several comorbidities coexist with COPD; some are related from a pathophysiological standpoint (pulmonary artery disease and malnutrition), whereas others are not directly related (anxiety, depression, osteoporosis, diabetes, obesity, anemia) [3]. Regardless of any pathophysiological association, comorbidities increase morbidity and economic burden while also significantly complicating the management of COPD [4,5]. Almost half of the financial burden was associated with comorbidities, while only 26% was attributed directly to COPD [5]. For this reason, the prevention and management of comorbid medical conditions is crucial in this population. Chronic kidney disease (CKD) is an important comorbidity of COPD that is not frequently discussed especially in older populations [6,7]. CKD is characterized by a glomerular filtration rate (GFR) <60 ml/min/1.73 m^2^ and/or some markers of kidney damage for more than three months based on the Kidney Disease: Improving Global Outcomes (KDIGO) guidelines [8]. A patient could have overt chronic renal failure (CRF) if the GFR is abnormal and creatinine serum value increases. To have concealed CRF, serum creatinine must be within the normal range while the GFR is abnormal [7].

Well-known systematic diseases such as vasculitides and autoimmune disorders have been found to affect both the lung and the kidney function [9]. Moreover, there is a collaboration between the lung and kidneys in physiological conditions to maintain acid-base balance and control blood pressure by the renin-angiotensin-aldosterone axis [10]. Chronic kidney disease may have a global prevalence of 11%-13% [11]. The correlation between COPD and CKD has not been thoroughly explored. This study is a narrative review of the association between COPD and CKD, with emphasis placed on the prevalence and risk factors for this comorbidity, its influence on mortality in comparison to either condition alone and the pathophysiological mechanisms by which COPD can promote the development of renal failure. To our knowledge, no relevant review on this has been published recently.

## Review

Methods

**Figure 1:**
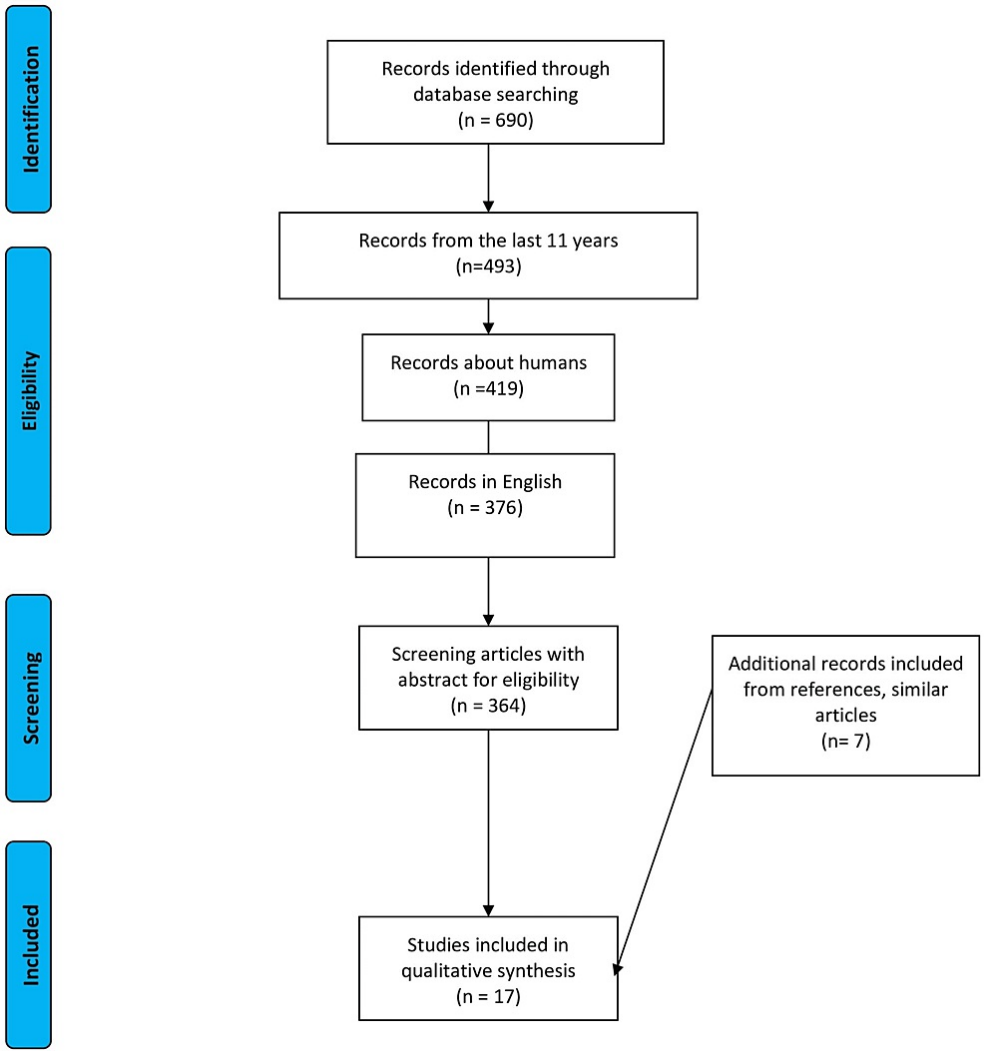
Selection flow chart

A thorough literature search via PubMed for relevant published studies was conducted. Keyword "COPD" and Medical Subject Headings (MeSH) term "pulmonary disease, chronic obstructive" in combination with the word "chronic renal failure or chronic renal disease" and MeSH term "kidney failure, chronic" were used. A total of 690 articles were identified. Inclusion and exclusion criteria were applied and studies that were written in any other language than English were removed. After screening, only clinical studies from the last 11 years were included. After screening, by going through the titles, abstract and full article, only 17 studies were left. Seven more studies were excluded as they discussed comorbidities of COPD in general and focused on other group of patients, such as vascular patients or diabetics. Ten relevant studies were included after thorough screening. Another seven studies were added from within references and similar articles for a more rounded result. The study was designed as a narrative review from its inception, so no quantitative data synthesis or meta-analysis was done. The literature search was conducted in a manner similar to systematic reviews for the sake of thoroughness and the selection flow chart provided showcases this. Since this work is intended to provide further information about COPD and renal failure, studies that illustrate this connection were collected. The results are presented in the selection flow chart in Figure 1.

Results

A total of 17 clinical studies were included in this review. Case-control studies, meta-analysis articles and other reviews were not used for this paper. There were a total of four observational studies, one clinical trial, 12 cohort studies, and two cross-sectional studies (in a single article). The entire sample of the included studies amounts to 13,176 COPD patients and 24,257 control patients without COPD and 98,633 participants not selected by COPD criteria.

Several studies showed the impact of COPD on renal failure mortality; others showed an increase in the prevalence of kidney failure in COPD patients. Most of the studies showed some correlation between lung function and the incidence or progression of kidney failure.

COPD and Renal Involvement

Renal involvement in COPD patients is a matter of discussion. Incalzi et al. gathered participants from 15 centers around Italy in an attempt to illustrate CRF prevalence in COPD patients over 65 years old [7]. They used 356 COPD and 290 aged-matched participants and discovered that 20.8% of COPD patients had concealed CRF versus 10% of non-COPD, while 22.2% had overt CRF versus 13.4% [6]. Moreover, they found a correlation between diabetes (odds ratio [OR], 1.96), hypoalbuminemia (OR, 2.83) and musculoskeletal disorders (OR, 1.78) with concealed CRF and a possible connection between diabetes (OR, 2.25) and body mass index (OR, 1.05) with overt CRF [6]. An increase in the CKD incidence in COPD patients was observed in a cohort study conducted in Taiwan with 11 years of follow-up (hazard ratio [HR], 1.61), as 183.38 additional cases per 10,000 persons were detected in the COPD group compared to the control group [12]. The mean patient age was 71 years; 7739 patients with COPD participated along with a control group in a 2:1 ratio. All the studies involved are summarized in Table 1.

**Table 1:** Prevalence of CKD in COPD patients COPD: chronic obstructive pulmonary disorder; CRF: chronic renal failure; CKD: chronic kidney disease; OR: odds ratio; HR: hazard ratio; eGFR: estimated glomerular filtration rate; ESRD: end-stage renal disease; FEV1: forced expiratory volume in the first second; CKDcys: chronic kidney disease based on cystatine; CKDcr: chronic kidney disease based on creatinine; FVC: forced vital capacity; eGFRcr: eGFR based on creatinine; eGFRcys: eGFR based on cystatine; AFL: airflow limitation; sTNF-R1: soluble tumor necrosis factor receptor 1; NGAL: neutrophil gelatinase-associated lipocalin

Author	Country of patients	Year of publication	Purpose of study	Number of patients	Type of study	Result/prevalence of CKD	Conclusion
Incalzi et al. [7]	Italy	2010	Prevalence of CRF in COPDpatients ³ 65	356 COPD, 290 non-COPD	Observational	20.8% concealed (10.0% without COPD), 22.2% overt (13.4% without COPD)	High prevalence was a comorbidity regardless of the creatinine values
Chen and Liao [12]	Taiwan	2016	Incidence of CKD in COPD patients	7739 COPD patients, 15,478 non-COPD	Case-cohort	HR: 1.61 overall (470.9 vs 287.52 per 10^4^ person-years)	Patients with COPD have a higher risk of CKD
AbdelHalim and AboElNaga [13]	Egypt	2016	Prevalence of CRF in COPD patients	136 COPD, 104 non-COPD	Cohort	19.85% concealed (1.92% non-COPD), 6.66% overt (0% non-COPD)	High prevalence of CRF in COPD patients
Elmahallawy and Qora [14]	Egypt	2013	Frequency of underdiagnosed renal failure	300 COPD and 300 control	Cohort	Normal renal function, concealed, overt in COPD patients 54%, 26%, 20%; in control, 78%, 10%, 12%	CRF is an important comorbidity and estimated GFR is needed for screening
Gjerde et al. [15]	Western Norway	2011	Frequency of undiagnosed renal failure in COPD patients and the relation with inflammatory markers	433 COPD (in 422, plasma creatinine measured), 233 non-COPD	Cohort	9.6% females with COPD and 5.1% males with COPD patients; GFR<60	Female sex, age, cachexia , and systemic inflammatory markers sTNF-R1 and NGAL associated with a higher risk of renal failure in COPD patients
Mapel and Marton [16]	New Mexico, USA	2013	Prevalence of renal or hepatic disease in COPD patients	2284 COPD and 5959 non-COPD	Cohort	Acute, chronic, and unspecified renal failure 1.40 vs 0.59, 2.89 vs 0.79, and 1.09 vs 0.44, respectively	COPD patients have an increased prevalence for renal, gallbladder or pancreatic diseases and are prescribed medications with potential adverse effects
Sumida et al. [17]	North Carolina, Mississippi, Minnesota and Maryland, USA	2017	Association of reduced lung function with ESRD and CKD	14,946	Prospective cohort	HR for CKD compared to high-normal 1.53, in mixed restrictive 1.42, obstructive 1.15, low-normal 1.08	Maybe there is pathophysiological contribution of lung function decline with the development of CKD
Yu et al. [18]	Australia		Association between lung and renal function	1298 normal renal function, 156 impaired	2 cross-sectional studies	Increase risk for renal impairment below 3.05, both for FEV1 and FVC in both studies	There was an association between obstructive lung function and reduced renal function
	Nanjing, China			4313 normal, 1511 Impaired			
Zaigham et al. [19]	Malmö, Sweden	2020	Low lung function early in life and development of CKD in the future	28,025	Prospective cohort	Q1 vs Q4, HR 1.46 in low FEV1 and HR 1.51 for FVC in men	Low FEV1 and FVC are a risk factor for future incident CKD in men, not women; FEV1/FVC <0.7 does not increase incidence for CKD in both men and women
Kim et al. [20]	Korea	2018	The role of lung function in the development of CKD	10,128 subjects	Retrospective cohort	FEV1/FVC <0.8, incidence of CKD 2.8%	Increased risk of CKD when airflow is limited; a decrease of 10% in FEV1 /FVC leads to 35% increase in the development of CKD
Yoshizawa et al. [21]	Japan	2015	Prevalence with eGFR based on creatinine and cystatin C levels	108 COPD, 73 non-COPD	Clinical trial	eGFRcr vs eGFRcys 31% vs 53% in COPD patients; 8% vs 15% non-COPD patients	Renal function of the Japanese should be measured with eGFRcystoo
Suzuki et al. [22]	Yamagata, Japan	2020	Mortality in COPD and CKD	1233 health checkup participants	Cohort	CKDcys 26.1% with AFL vs 16.2% without AFL	Higher prevalence with CKDcys in AFL, but not with CKDcr

A study from Egypt by Abdelhalim and AboElNaga involving 136 COPD patients and 104 control subjects provided slightly different results [13]. The percentage for concealed and overt CRF in COPD patients was 19.85% and 6.62%, respectively, whereas in the control group, it was 1.92% and 0%, respectively. The study found a greater prevalence of CRF in patients with a more advanced stage of COPD (Global Initiative for Chronic Obstructive Lung Disease, or GOLD, stages C and D) [13]. Another study by Elmahallawy and Qora involving 600 patients (300 COPD and 300 control) found a higher prevalence of CRF [14]. In the control group, normal renal function was present in 78% of the sample, while 10% of the participants had concealed renal failure and 12% overt, whereas in the COPD group, the prevalence was 54%, 26%, and 20%, respectively. Although there were similarities in the two studies regarding the concealed percentage of CRF, they showed a great difference for the overt group. These differences could be explained by the selection of patients and the estimation of GFR.

In a different study in western Norway, 433 COPD patients and 233 non-COPD were analyzed, and it was found that undiagnosed renal failure was 6.9% and 0.8%, respectively [15]. Almost 7% could be a lower number than the actual one in a real population, but this happened because study participants were younger (40-76 years old). Subjects with already known renal failure were excluded.

In a larger study, where 2284 COPD patients and 5959 matched non-COPD patients were included, it was found that COPD patients had a higher prevalence for acute, chronic and unspecified renal failure [16]. The results per 100 persons were 1.4 versus 0.59 for acute, 2.89 versus 0.79 for chronic and 1.09 versus 0.44 for unspecified. Another interesting factor of this study was that 29% of COPD patients had at least one abnormal renal test, whereas, in controls, only 17% had one abnormal renal test [16].

In the Atherosclerosis Risk in Communities study, 14,946 participants aged 45-64 years were included [17]. The median follow-up was 23.6 years. It showed that participants with restrictive and obstructive lung function patterns had HRs of 2.03 and 1.47, respectively, for the development of the end-stage renal disease compared with those with normal lung function.

One study illustrated that patients with obstructive respiratory patterns were at increased risk of renal function decline [18]. The study was based on two separate cross-sectional studies, one in Australia and one in China, and its main finding was that people with forced expiratory volume in the first second (FEV1) and forced vital capacity (FVC) below 3.05 L were more likely to have reduced renal function. Another study in Malmö, Sweden, concluded that men with low FEV1 and FVC had a higher risk for CKD than women [19]. No association was found between COPD (defined by at least one measurement of FEV1/FVC <70) and the incidence of CKD. A different study did however find a significant association [20]. During the follow-up period of five years, participants with FEV1/FVC <0.8 had a higher incidence of CKD (HR, 1.454). This cohort comprised 10,128 patients in total.

Regarding the impact of COPD on the mortality of patients with CKD, it was found that the patients with COPD had a higher risk of mortality (HR, 1.22) compared to the control group without COPD [23], as it is shown in Table 2. However, in a study where 404 subjects from every GFR category were involved, it was found that the restrictive lung dysfunction resulted in increased mortality risk (HR, 1.80), whereas in the obstructive lung impairment group, the mortality risk was not statically significant [24]. In the same study, it was found that the prevalence for the obstructive disease was different between the GFR groups (from 6% in G1 to 11% in G5) with a similar pattern being observed in the restrictive group (from 9% in G1 to 36% in G5). Selection bias could be responsible for these differences between studies. Table 2 showcases all the studies regarding the impact of COPD on the mortality of patients with CKD.

**Table 2:** Impact on mortality in patients with both COPD and CKD COPD: chronic obstructive pulmonary disorder; CRF: chronic renal failure; CKD: chronic kidney disease; OR: odds ratio; HR: hazard ratio; eGFR: estimated glomerular filtration rate; ESRD: end-stage renal disease; CKDcys: chronic kidney disease based on cystatine

Author	Country of patients	Year of publication	Purpose of study	Number of patients	Type of study	Result/mortality with CKD	Conclusion
Suzuki et al. [22]	Yamagata, Japan	2020	Mortality in COPD and CKD	1233 health checkup participants	Cohort	Mortality HR: 2.94 when they have CKD and airflow limitation, 1.29 if only airflow limitation, 1.45 if CKDcys alone	When the two diseases coexist, there is an important risk factor for mortality
Lai et al. [23]	Taiwan	2018	Impact of COPD in advanced CKD	33,399 enrolled, but two similar subgroups with 1820 patients each were created	Cohort	HR 1.22 for mortality	COPD increases the mortality in elderly and males, but not the risk of ESRD
Mukai et al. [24]	Huddinge, Sweden	2018	Correlation of mortality among individuals with normal to severe GFR with lung function	404 individuals with lung function: normal 252, obstructive 42, restrictive 110	Observational	Restrictive lung dysfunction mortality risk sHR: 1.8; no statistically significant obstructive mortality risk. The prevalence of obstructive disease 6% in G1 vs 11% in G5; restrictive 9% in G1 vs 36% in G5	Lung dysfunction is associated with renal function decline
Fabbian et al. [25]	Ferrara, Italy	2016	Relationship between CKD, acute kidney injury and in-hospital mortality in patients admitted for COPD exacerbation	7073 exacerbation COPD patients	Observational retrospective	10.9% CKD prevalence; 5% acute kidney injury	Acute kidney injury is an important factor for in-hospital mortality (OR, 3.849), especially in older males with other comorbidities
Fedeli et al. [26]	Veneto, Italy	2017	Relationship between comorbidities, CKD and mortality in patients with COPD diagnosis	27,272 patients	Cohort	CKD mortality HR: 1.36	Comorbidities and CKD affect the survival of COPD patients
Trudzinski et al. [27]	Germany	2019	Relationship between COPD, CKD, eGFR outcomes and mortality	2274 patients with CKD 161 (7.1%)	Cohort	CKD HR: 2.3	CKD is a relevant comorbidity in COPD patients that affects mortality

A study in Italy regarding COPD patients hospitalized with exacerbations found that acute kidney injury was an important factor in inpatient mortality (OR:3.849) [25]. The prevalence of CKD and acute kidney injury in this sample was 10.9% and 5%, respectively. Another study in Italy found a hazard ratio for mortality of 1.36 when COPD and CKD coexist, whereas a study in Germany with 2274 COPD patients, of whom 7.1% had CKD, found a hazard ratio of 2.3 [26,27]. In both studies, the CKD patients were older than the rest of the participants, but both studies performed Cox regression analysis for age and other confounders after which the association retained its significance.

Another possible explanation for the discrepancies between study results may be the way GFR is estimated. A group from Japan proposed in 2015 that for COPD patients in the Japanese population, renal function could be measured more accurately by calculating GFR based on cystatin levels and not only creatinine (eGFRcr) [21]. This study showed that the percentage of CKD prevalence rose when measured with GFR based on cystatine (eGFRcys) from 31% to 53% in the COPD group and from 8% to 15% in the non-COPD group. The Yamagata study used these measurements and found that people with FEV1 70%, of the normal value, in spirometry were more likely to be diagnosed with CKD if renal function was assessed by eGFRcys (26.1%) than with eGFRcr (10.9%) and with a higher risk of mortality when both measurements were indicative of CKD [22].

Discussion

**Figure 2:**
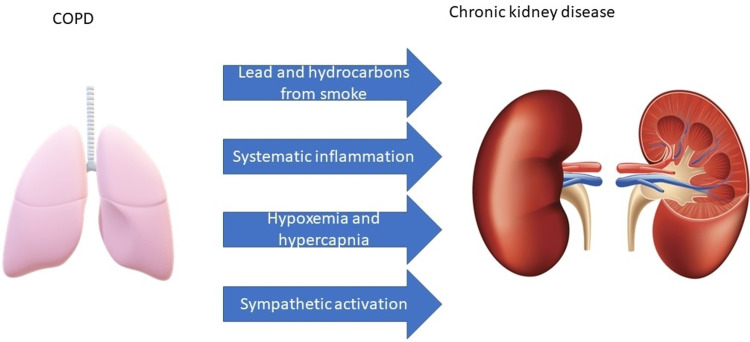
Risk factors that worsen renal function in COPD COPD: chronic obstructive pulmonary disease

From the definition of COPD, it is obvious that noxious gases play a vital role in the pathogenesis of the disease. Nicotine is not a strong contributor to lung tissue damage in COPD, but it may affect renal function to a greater extent. It activates the sympathetic autonomic system causing an increase in blood pressure [8]. This could worsen any nephropathy that a patient already has. It may also reduce the activity of enzymes that protect cells from oxidative stress such as superoxide dismutase and catalase in the kidney. The components that are usually found in smoke could induce endothelial dysfunction and atherosclerosis via the reactive oxygen elements, further contributing to renal dysfunction [5]. Risk factors that worsen renal function in COPD patients are illustrated in Figure 2.

Hypoxia can also result in renal damage [28]. Certain cells of the kidney have a particularly high oxygen demand (specifically the cells lining the proximal convoluted tubules) and even subclinical hypoxia could result in apoptosis of these cells and tissue fibrosis [28]. Hypercapnia in COPD patients can cause renal afferent arteriolar vasoconstriction, reduced renal blood flow and activation of the adrenergic pathway [29].

Another important factor that plays a significant role in the worsening of renal function in COPD patients is age [5]. These are diseases that primarily present in individuals with advanced age and disease progression may be accelerated in the geriatric population as senescent cells may be unable to respond effectively to local tissue insults such as the presence of smoke particles or inflammation. The mechanisms of tissue repair and other homeostatic pathways become less effective with advancing age as well [5]. Other comorbid conditions (such as atrial fibrillation and congestive heart failure) commonly afflicting geriatric patients also contribute to the renal function decline.

Systematic inflammation and its associated prothrombotic state could also contribute to kidney damage in COPD patients. In an observational study in 2017, Zeng et al. analyzed 106 COPD and 106 non-COPD patients [30]. The prevalence of concealed CRF (19.28%) was higher in COPD patients, whereas in the control group, it was 7.55%. An association between elevated CRP and D-dimer levels (ORs, 1.252 and 1.095, respectively) was found for concealed CKF. Interestingly, an inverse association was found between CKD and levels of ADAMTS-13 (OR, 0.858), suggesting that this enzyme may have a role in the preservation of normal renal function [30]. In the same vein, another study from 2011 found that that female sex, older age, cachexia, and plasma concentrations of the systematic inflammatory markers soluble tumor necrosis factor receptor 1 (sTNF-R1) and neutrophil gelatinase-associated lipocalin (NGAL) were associated with CKD in COPD patients [15].

Elderly individuals have a higher medication burden and medication toxicity may significantly contribute to the pathogenesis of CKD [31]. Besides the damage to the kidneys that nephrotoxic drugs could cause, patients with CKD are at an increased risk of adverse effects of water-soluble drugs that are excreted in the urine.

It would be best at this point to acknowledge the limitations of this review. First of all, it was based mainly on observational studies that by definition can establish correlation but not causation. The prevalence of CKD in COPD patients may be different between countries and population subgroups studied. Selection bias and low statistical power (a consequence of small sample size) may be responsible for many of the discrepancies between studies. Prospective studies with matched control groups and larger samples are required to estimate the extent to which CKD increases mortality in COPD patients. eGFR calculated using cystatin instead of creatinine should also be included in more studies to determine whether it is indeed a more sensitive indicator of renal dysfunction in COPD. Although the exact mechanisms for the connection are not well understood right now, this work highlights the association between CKD and COPD and its clinical implications while also providing the groundwork for further research into the matter.

## Conclusions

During the past decade, a strong association between COPD and CKD has emerged. CKD is more common in individuals suffering from COPD compared to age-matched controls, and COPD patients with comorbid CKD are at a greater risk of adverse outcomes. Comorbid CKD is correlated with increased mortality in this group and it would be of great interest to examine whether it would increase the frequency of exacerbations and the requirements for inpatient treatment as well. Certain discrepancies between study findings necessitate further research with prospective cohort studies characterized by larger samples and uniform selection criteria. Because of the association discussed in this work, clinicians should frequently screen COPD patients for CKD and avoid prescribing nephrotoxic medications unless absolutely necessary due to the risk of accelerating the progression of CKD.
